# First Multi-Organ Full-Length Transcriptome of Tree Fern *Alsophila spinulosa* Highlights the Stress-Resistant and Light-Adapted Genes

**DOI:** 10.3389/fgene.2021.784546

**Published:** 2022-02-04

**Authors:** Yongfeng Hong, Zhen Wang, Minghui Li, Yingjuan Su, Ting Wang

**Affiliations:** ^1^ School of Life Sciences, Sun Yat-sen University, Guangzhou, China; ^2^ Research Institute of Sun Yat-sen University in Shenzhen, Shenzhen, China; ^3^ College of Life Sciences, South China Agricultural University, Guangzhou, China

**Keywords:** *Alsophila spinulosa*, tree fern, full-length transcriptome, stress resistance, light adaptation

## Abstract

*Alsophila spinulosa*, a relict tree fern, is a valuable plant for investigating environmental adaptations. Its genetic resources, however, are scarce. We used the PacBio and Illumina platforms to sequence the polyadenylated RNA of *A. spinulosa* root, rachis, and pinna, yielding 125,758, 89,107, and 89,332 unigenes, respectively. Combining the unigenes from three organs yielded a non-redundant reference transcriptome with 278,357 unigenes and N50 of 4141 bp, which were further reconstructed into 38,470 UniTransModels. According to functional annotation, pentatricopeptide repeat genes and retrotransposon-encoded polyprotein genes are the most abundant unigenes. Clean reads mapping to the full-length transcriptome is used to assess the expression of unigenes. The stress-induced *ASR* genes are highly expressed in all three organs, which is validated by qRT-PCR. The organ-specific upregulated genes are enriched for pathways involved in stress response, secondary metabolites, and photosynthesis. Genes for five types of photoreceptors, CRY signaling pathway, ABA biosynthesis and transduction pathway, and stomatal movement-related ion channel/transporter are profiled using the high-quality unigenes. The gene expression pattern coincides with the previously identified stomatal characteristics of fern. This study is the first multi-organ full-length transcriptome report of a tree fern species, the abundant genetic resources and comprehensive analysis of *A. spinulosa*, which provides the groundwork for future tree fern research.

## Introduction

*Alsophila spinulosa* is a palm-like tree fern with a large erect rhizome, belonging to order Cyatheales, family Cyatheaceae (PPG I, 2016). Tree ferns had originated independently from the distinct lineages and were well established during the late Carboniferous to Triassic periods, but many of them became extinct in the late Permian period (Large and Braggins, 2004; Cleal and Thomas, 2019). To date, the majority of extant tree ferns are composed of Cyatheales originating from the Jurassic to Cretaceous periods (Large and Braggins, 2004; Smith et al., 2008). Cyatheales is not only the representative taxon of relict tree ferns but also the second largest fern clade (monilophytes) (PPG I, 2016). The family Cyatheaceae, which accounts for 90% of the species within Cyatheales, has undergone hyper radiation and diversification (Janssen et al., 2008; Loiseau et al., 2020). Among the genus *Alsophila* with ca. 230 species, a total of 68 species are listed on the International Union for Conservation of Nature (IUCN) Red List (IUCN, 2010). The relict tree ferns are valuable species for exploring adaptive evolution, particularly in stress resistance and light adaptation. Among the three major lineages within the core leptosporangiates (Janssen et al., 2008), the full-length genomic and transcriptomic researches have focused on heterosporous ferns (Li et al., 2018) and polypod ferns (Sun et al., 2018; Marchant et al., 2019; Yan et al., 2019), whereas tree ferns are the most neglected lineage. Only a few transcriptomes of tree fern species have been covered in large-scale phylogenetic analysis, including two relatively low-quality transcriptomes of *A. spinulosa* (Dong et al., 2019; One Thousand Plant Transcriptomes Initiative et al., 2019).

*Alsophila spinulosa* is the most representative tree fern among three species of the genus *Alsophila* distributed in mainland China (Dong et al., 2019; Loiseau et al., 2020). The tree fern, especially the stem part, has been utilized for traditional medicine, from which a variety of flavonoids and phenolic compounds have been characterized (Chiang et al., 1994). With trunks up to 5–15 m tall and fronds up to 2–3 m long (Zhang and Nishida, 2013), the plant is well adapted to moderate to shaded niches in humid forests (Zhiying et al., 1990; Nagano and Suzuki, 2007; Chiu et al., 2015). Plants at different levels of the forest hierarchy have evolved their photoreceptors to adapt to varying light conditions (Mathews, 2006). The ability of ferns to thrive in angiosperm-dominated forests is closely linked to the evolution of their photoreceptors (Schneider et al., 2004). Plants have five common types of photoreceptors: phytochrome (PHY) for red/far-red lights, phototropin (PHOT), cryptochrome (CRY), ZEITLUPE (ZTL) for blue/UV-A lights, and UVR8 for UV-B light (Bae and Choi, 2008). In addition, neochrome (NEO), a chimeric photoreceptor that combines PHY and PHOT, has been found in a small number of ferns (Kawai et al., 2003; Yang et al., 2010) but not in tree ferns. The *CRY* genes of Polypodiales species occurred in lineage-specific duplication events, which are probably related to their rapid stomatal opening under blue light (Cai et al., 2020). The CRY signaling pathway-related genes that indirectly trigger stomatal opening have been demonstrated in *Arabidopsis thaliana* (Ando et al., 2013) and profiled in a polypod fern, *Nephrolepis exaltata* (Cai et al., 2020), but not for tree fern.

The regulation of stomatal opening and closing, which maintains water balance between photosynthesis and transpiration, is a crucial step in plant evolution (Jones, 1998). Ferns are the highly diversified lineage of basal plants whose stomatal regulation mechanism is still debated. Stomatal closure in angiosperms is actively driven by abscisic acid (ABA) (Lim et al., 2015), whereas it is passively induced in most ferns by hydraulics (McAdam and Brodribb, 2012). In angiosperms, SNF1-related protein kinase 2 (SnRK2) downstream of ABA signaling plays a core role in promoting stomatal closure (Hsu et al., 2018). On one hand, open stomata 1 (OST1, also known as SnRK2.6) phosphorylates slow anion channel 1 (SLAC1), leading to stomatal closure (Vahisalu et al., 2008). Nitric oxide (NO) generation, on the other hand, is an essential signal factor for stomatal closure via the SnRK2 signaling pathway (Gong et al., 2021). Nitric oxide synthase and nitrate reductase are involved in ABA-induced NO synthesis to close stomata (Neill et al., 2002; Bright et al., 2006). Nevertheless, in ferns, the ABA-SnRK2 signaling pathway regulates spore dormancy and sex determination rather than stomatal closure (McAdam et al., 2016). Physiological studies have revealed that SnRK2 in ferns is unable to mediate stomatal closure because it cannot generate NO (Gong et al., 2021). The combination of these two mechanisms in ferns, rapid stomatal opening and passive stomatal closure, may optimize the photosynthetic capacity and confer the understory adaptation (Way and Pearcy, 2012; Cai et al., 2020). However, the ABA synthesis and signaling pathways in tree fern have yet to be thoroughly studied. In addition, the abscisic acid-, stress-, and ripening-induced (*ASR*) gene, which is upregulated by ABA, has an important role in crop plant resistance to water deficit stress (Li et al., 2017), but it has not been studied in ferns.

*Alsophila spinulosa* is a diploid organism with a chromosome count of 138 (2n = 138) (Mehra, 1955) and a predicted genome of 6.0 G by flow cytometry (Huang et al., 2020). The genome of the model fern, *Ceratopteris richardii,* revealed that the giant genome consists of a large fraction of LTR retrotransposons (Marchant et al., 2019). Retrotransposons are triggered in response to stressors such as abscisic acid (He et al., 2010), fungal infection (Sabot et al., 2006), UV light (Ramallo et al., 2008), drought (Lu et al., 2012), and heavy metals (Castrillo et al., 2013), which improves stress tolerance by increasing the plasticity of genome (Wessler, 1996; Piegu et al., 2006). The retrotransposon-encoded polyprotein is responsible for transposition and increases the genome size (Grandbastien et al., 1989), but their transcriptional levels in ferns remain unclear. Although it is challenging to sequence the whole genome of *A. spinulosa*, its chloroplast genome has been defined (Gao et al., 2009). *Adiantum capillus-veneris*, a polypod fern, exhibits high levels of post-translational processing, including site editing, intron splicing, and cleavage of polycistronic transcripts (Wolf et al., 2004). A substantial number of RNA editing sites were also predicted in the chloroplast genome of *A. spinulosa* (Gao et al., 2009). RNA sequencing was employed to confirm chloroplast post-transcriptional processing (Wu et al., 2021). The fusion transcripts have been reported in the chloroplast of *Adiantum capillus-veneris* by Sanger sequencing (Wolf et al., 2004). Full-length RNA sequencing can reveal the actual transcripts in the chloroplast, especially the long transcripts. The pentatricopeptide repeat (PPR) proteins, the largest family in the plant genome (Aubourg et al., 2000), contain 31–36 amino acid tandem repeat modules, which are responsible for post-transcriptional processing of organellar genes in terrestrial plants (Manna, 2015). As a result, PPR proteins regulate organelle gene expression (Meierhoff et al., 2003). Plants respond to abiotic stressors through post-transcriptional processing of chloroplast RNA (cpRNA) (Dinh et al., 2016), implying that PPR proteins may play a role in environmental adaptation. However, the fusion transcripts of chloroplast and the abundance of *PPR* genes in tree fern are still unknown.

**FIGURE 1:**
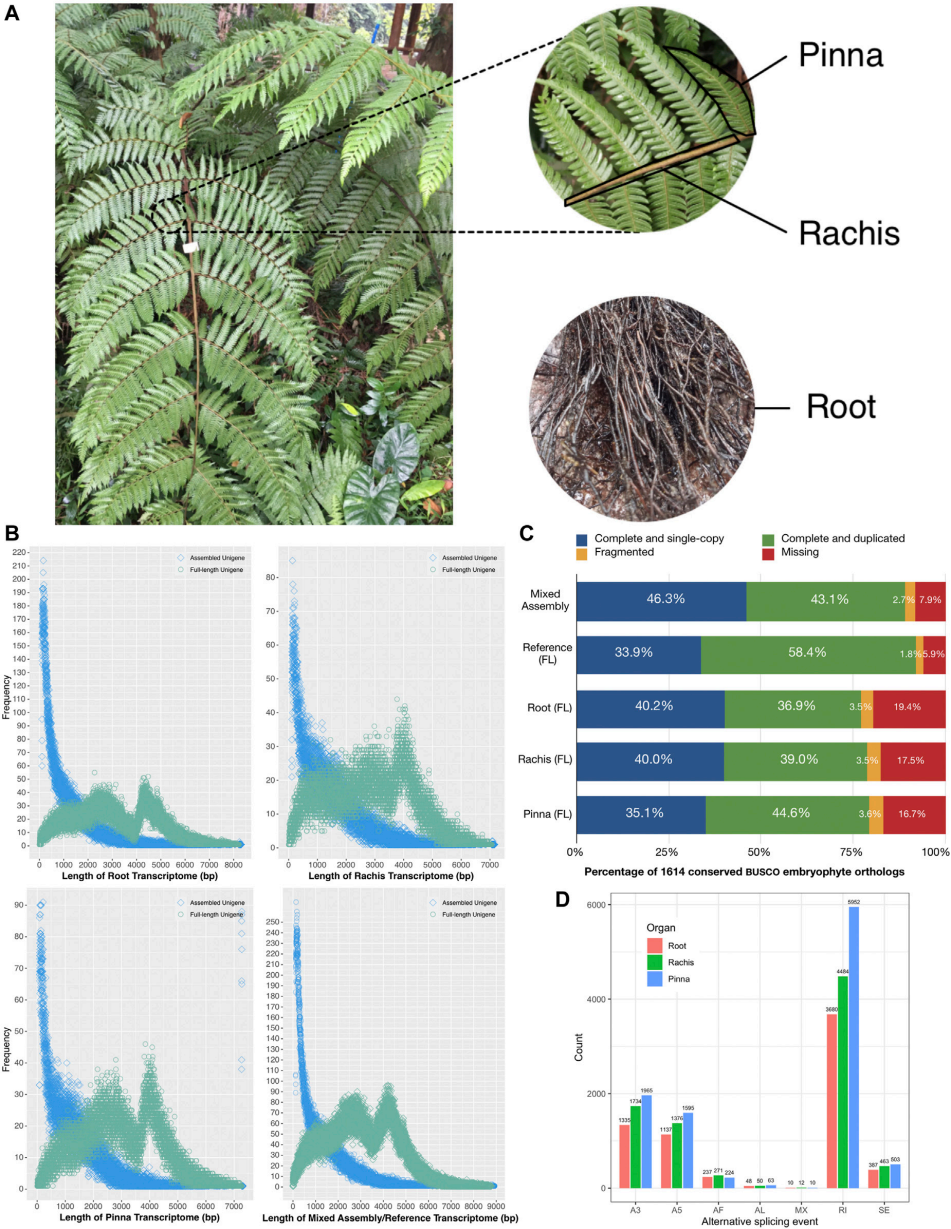
Schematic diagram of *Alsophila spinulosa* transcriptome quality assessment via visualizing length distribution, BUSCO evaluation, and AS events detection. **(A)** Schematic diagram of root, rachis, and pinna of *A. spinulosa*. **(B)** Frequency of unigenes length in the root, rachis, pinna, mixed assembly, and FL reference transcriptomes. **(C)** BUSCO evaluation of the root, rachis, pinna, mixed assembly, and FL reference transcriptomes. **(D)** The number of seven types of AS event in three organs.

The pinna (leaflet) of *A. spinulosa* is the photosynthetic organ (Figure 1A). The rachis is the middle stems of leaves, which play a role in the control of water transduction in the xylem. The root is not only for absorbing water and nutrients but also has a supportive and protective role (Nagano and Suzuki, 2007). To obtain a comprehensive full-length (FL) transcriptome, Pacific Biosciences (PacBio) long-read technology was used to sequence these three representative organs of *A. spinulosa* in their natural environment, which were calibrated by short reads from Illumina sequencing. The FL transcriptomes were mined for the following purposes: 1) quality assessment; 2) functional annotation and identification of the fusion transcripts in chloroplast; 3) structural prediction; 4) expression quantification and extraction of highly expressed genes; 5) differential gene expression between organs and enrichment analysis of organ-specific upregulated genes; and 6) characterization of genes involved in light adaptation and stomatal regulation. For the first time, the FL transcriptome highlights the stress-resistant and light-adapted genes in *A. spinulosa*. It serves as a valuable molecular resource for future in-depth studies of tree fern species.

## Materials and Methods

### Plant Material and Ribonucleic Acid Extraction

The fresh root, rachis, and pinna of a single *A. spinulosa* plant were gathered in the early afternoon of December 30, 2018, at South China Botanical Garden (23°11′14.3″N, 113°22′03.5″E), Guangzhou, Guangdong Province, P. R. China. The samples were washed immediately and dried up before being immersed in RNAlater solution (BioTeke, Shanghai, China). The samples of three organs were preserved at −20°C until RNA extraction. Total RNA of each sample was extracted using the RNeasy Plus Mini kit (QIAGEN, Hilden, Germany), and then each sample went through the following steps individually. RNA concentration and quantity were assessed by Qubit 2.0 fluorometer (Thermo Fisher Scientific, Waltham, MA, USA). RNA integrity number (RIN) was evaluated by Agilent 2,100 bioanalyzer (Agilent Technologies, Santa Clara, CA, United States). The RNA quality criteria for the construction of the SMRTbell library and the NGS library were concentration ≥300 ng/μL, quantity ≥4 μg, and RIN ≥7.0. For each organ, a total RNA sample that met the requirements was chosen for subsequent steps.

### Illumina Ribonucleic Acid-Seq and *De Novo* Transcriptomes Assembly

Before random RNA fragmentation, the RNA with poly-A tail of each organ was isolated from the total RNA by Oligo dT beads. Then the NEBNext Ultra RNA Library Prep Kit for Illumina (New England Biolabs, Ipswich, MA, United States) was used to prepare the NGS library. On the NovaSeq 6,000 System (Illumina, San Diego, CA, United States), the library of each organ was sequenced separately with 300 bp per paired-end read. The following raw reads were filtered by Trimmomatic v0.39 (Bolger et al., 2014): the reads containing adapter, the reads with more than 10% unknown bases, and the reads with more than 50% low-quality bases (Phred quality score <20). The clean reads of each generated organ were used for self-assembly by Trinity v2.4.0 (Haas et al., 2013), with the minimum *k*-mer coverage set to 3. Subsequently, the *de novo* assembly sequences were clustered to obtain the unigenes by Corset v1.05 (Davidson and Oshlack, 2014). The three-organ clean reads were combined for hybrid assembly in the same way as above.

### PacBio Iso-Seq and Full-Length Transcriptomes Building

The mRNA of each organ was enriched by Oligo dT beads and reverse-transcribed into cDNA using the SMARTer PCR cDNA Synthesis Kit (Clontech, Mountain View, CA, United States). The >4 kb cDNA was then partitioned by BluePippin Size Selection System (Sage Science, Beverly, MA, United States) and amplified using PCR. The unfiltered cDNA and >4 kb cDNA fractions were mixed in a mole ratio of 1:1. The mixed cDNA was used for preparing the SMRTbell library by the SMRTBell Template Prep Kit (Pacific Biosciences, Menlo Park, CA, United States). Finally, the library of each organ was sequenced independently on a single Sequel II System SMRT Cell (Pacific Biosciences).

The polymerase reads of each organ were processed through SMRT Link v6.0. The subreads were created by splitting the polymerase reads and removing the adapters. The self-correction circular consensus sequences (CCS) were obtained from the consensus sequences of subreads in a single zero-mode wave guide (ZMW) with at least two full passes. The CCS were then categorized as full-length non-chimeric (FLNC) reads, full-length chimeric reads, and non-full length non-chimeric (NFLNC) reads according to the presence or absence of 5′ primer, 3′ primer, and poly-A tail. FLNC were grouped into the unpolished cluster consensus reads by isoform-level clustering (ICE), following that the reads were polished with NFLNC reads. The polished cluster consensus reads from the Iso-Seq pipeline were fed into the hybrid error correction process, meaning each sequence was proofread based on the high-accuracy RNA-Seq clean reads by LoRDEC v0.7 (Salmela and Rivals, 2014). The corrected consensus sequences reduced redundancy with the threshold of 95% similarity using CD-HIT v4.6.8 (Li and Godzik, 2006). As a result, the non-redundant FL unigenes were obtained for each organ, which were further classified as high-quality (HQ) and low-quality (LQ) unigenes based on whether the consensus accuracy reached 99%. The three-organ FL unigenes were combined and the redundant unigenes were clustered using CD-HIT with the same criterion above.

The non-redundant three-organ FL unigenes were further reconstructed into UniTransModels using Cogent v 8.0.0 (https://github.com/Magdoll/Cogent) with default parameters. In brief, the FL unigenes were clustered into the gene family based on their k-mer similarity, and each family was reconstructed into the UniTransModels by de Bruijn graph method. Then, the FL unigenes of three organs were individually mapped to the UniTransModels by GMAP v 2021–08-25 (Wu and Watanabe, 2005). Seven types of alternative splicing (AS) events were detected in each organ by SUPPA v 2.3.0 (Trincado et al., 2018), including the alternative 5′ (A5) and 3′ (A3) splice sites, alternative first (AF) and last (AL) exons, mutually exclusive (MX) exons, retained intron (RI), and skipping exon (SE) events. To validate the completeness of the assembled and FL transcriptomes, BUSCO v5.0.0 (Simão et al., 2015) was employed by searching the 1,614 embryophyte orthologues.

### Full-Length Transcriptomes Function Annotation

To decode the gene function as broadly as possible, the FL unigenes were annotated to NCBI NT (Nucleotides database) by BLAST v2.7.1+ (Altschul et al., 1990) and NCBI NR (Non-redundant database), KOG (euKaryotic Orthologous Groups), Swiss-Prot, and KEGG (Kyoto Encyclopedia of Genes and Genomes) by Diamond v0.8.36 BLASTX (Buchfink et al., 2015) with an E-value of < 1e-5. The protein families of the FL unigenes were detected by searching the Pfam databases using hmmscan of the HMMER v3.1 package (Finn et al., 2011). GO (Gene Ontology) annotation was generated by mapping the Pfam entries to GO terms using an in-house script based on pfam2go (http://current.geneontology.org/ontology/external2go/pfam2go).

### cpRNA, Coding Sequences, Transcription Factors, Simple Sequence Repeats, and LncRNA Prediction

The candidates of cpRNA were retrieved from the HQ FL unigenes that aligned to the complete chloroplast genome of *A. spinulosa* in NT database (GenBank: FJ556581). These candidates of cpRNA were mapped back to the chloroplast genome of *A. spinulosa* by Geneious v9 (Kearse et al., 2012) for visualizing the transcription using the most sensitive mode. The coding sequences (CDS) of FL unigenes were predicted by ANGEL v2.4 (Shimizu et al., 2006) with the default tolerant mode and the threshold of >50 amino acid residues. Transcription factors (TF) were predicted by iTAK v1.7 (Zheng et al., 2016). Simple sequence repeats (SSR) were detected using MISA v1.0 with the following minimum repeat times: mono-10, di-6, tri-5, tetra-5, penta-5, and hexa-5. Four software, CNCI v2 (Sun et al., 2013), CPC v0.9 (Kong et al., 2007), PfamScan v0.9 (Finn et al., 2016), and PLEK v1.2 (Li et al., 2014), were utilized for predicting the candidates of long non-coding RNA (LncRNA). The intersection of four sets of presumptive LncRNA with at least 200 bp were considered as real LncRNA.

### Genes Expression Quantification and Differentially Expressed Gene Enrichment

The RNA-Seq clean reads from each organ were mapped to the non-redundant unigenes combined by three organs using Bowtie2 (Langmead and Salzberg, 2012) with end-to-end alignment mode. On the one hand, the number of the mapped reads for each FL unigenes was counted by RSEM (Li and Dewey, 2011) to obtain the read count values, which were then normalized to fragments per kilobase million (FPKM) to assess gene expression in each organ. The read count values, on the other hand, were fed into the DEGseq R package (Anders and Huber, 2010) for differentially expressed gene (DEG) analysis between pairwise organs, which normalizes gene expression using the trimmed mean of M-values (TMM) method. Each pair of DEGs was subjected to Fisher’s exact test and then adjusted by Benjamini-Hochberg (BH) procedure. The significant DEGs were screened with the criterion of |log2 (fold change)| >1 and *q*-value <0.005.

The GO term and KEGG pathway enrichment analysis were performed in the clusterProfiler R package (Yu et al., 2012). The GO and KEGG annotation of the non-redundant FL unigenes was used as the background. The overlapping upregulated genes of each organ (root *vs.* rachis overlap root *vs.* pinna, rachis *vs.* root overlap rachis *vs.* pinna, and pinna *vs.* root overlap pinna *vs.* rachis) were used as the foreground. Each GO term/KEGG pathway was verified by Fisher’s exact test with BH adjustment, and the enriched GO term/KEGG pathway with default *q*-value threshold (<0.2) was considered significant.

### Photoreceptor Genes Identification and Abscisic Acid, Stress, and Ripening-Induced Genes Validation

Based on the Swiss-Prot annotation of HQ FL unigenes, the candidate photoreceptor genes in *A. spinulosa* were retrieved (*PHY*, *PHOT*, *CRY*, *ZTL*, and *UVR8*), and the non-expressing genes were filtered out using the FPKM >1 criterion in at least one organ. After that, the CDS sequences corresponding to these photoreceptor genes were retrieved from the ANGEL prediction results and de-redundant using CD-HIT with the cutoff of <95% similarity. The CDS of the photoreceptor genes was verified by checking the typical protein domains in Pfam database annotation. To explore the phylogeny of *CRY* genes, CRY protein sequences of representative species in lycophyte (one species), monilophyte (16 species), and spermatophyte (12 species) were obtained from the OneKP database (One Thousand Plant Transcriptomes Initiative et al., 2019), the transcriptome datasets of 69 ferns (Shen et al., 2017), and several model plant databases following the protocol of Cai et al. (2020). The 93 CRY protein sequences from 30 plants including *A. spinulosa* were aligned by MAFFT v7.475 using automatic mode. The conserved blocks of alignment were screened by Gblock v0.91 (Castresana, 2000). The best-fit model of amino acid substitution was selected using IQ-tree v2.0.3 (Minh et al., 2020) for constructing the maximum likelihood tree with 1,000 bootstraps. *Dendrolycopodium obscurum*, a species of lycophyte, was set as the outgroup. Members of *CRY* were classified according to Cai et al. (2020).

Identification and de-redundancy of the *ASR* gene family were carried out in the same way as photoreceptor genes. Protein motifs of *ASR* gene were detected by MEME suite v5.3.3 (Bailey et al., 2015). The *ASR* protein sequences of three species of Cyatheales (*Culcita macrocarpa*, *Plagiogyria japonica*, *Thyrsopteris elegans*), three species of Polypodiales (*Woodsia scopulina*, *Cystopteris utahensis*, *Pityrogramma trifoliata*), and *Selaginella moellendorffii* were retrieved from OneKP database by BLASTP using the ASR protein of *A. spinulosa* as the query. The 30 ASR protein sequences, including *A. spinulosa,* were used to construct the phylogenetic tree by IQ-tree v2.0.3 with *S. moellendorffii* as the outgroup. One of the *ASR* FL unigenes was used for validating the expression through qRT-PCR. Briefly, total RNA of root, rachis, and pinna was extracted as described above and then reverse-transcribed into cDNA templates by HiScript III RT SuperMix for qPCR Kit (Vazyme, Nanjing, China). The primer pairs were designed by Primer3Plus (Untergasser et al., 2007). The qRT-PCR assay was performed in triplicate using ChamQ SYBR Color qPCR Master Mix Kit (Vazyme, Nanjing, China). The PCR procedures consisted of 95°C 30s, 95°C 10 s, 60°C 30 s, for 40 replicates and verified by the standard melting curve. The root was used as control sample and the β-actin FL unigene was used as reference gene. The relative expression of *ASR* unigene in rachis and pinna was calculated by 2^–∆∆Ct^ method.

### Stomatal Movement-Related Pathways Profiling

The stomatal opening-related genes regulated by CRY were summarized from the “circadian rhythm – plant” KEGG pathway (ko04712) and the experimental evidence of Ando et al*.* (2013). The genes for the ABA biosynthesis pathway were derived from the “carotenoid biosynthesis” KEGG pathway (ko00906). The ABA signal transduction pathway associated genes were extracted from “plant hormone signal transduction” KEGG pathway (ko04075) and the experimental report of Gong et al*.* (2021). Stomatal movement-related ion channel and transporter genes were obtained from the review of (Kollist et al*.*, 2014), Saito and Uozumi (2019). All genes in the above pathway were searched from the Swiss-Prot annotation of HQ FL unigenes, and the non-expressed genes were filtered out with a threshold value of FPKM >0.3 in at least one organ. The CDS sequences corresponding to these genes were de-redundant using CD-HIT with the <95% similarity threshold. The expression profiles were visualized using the R package heatmap.

## Result

### PacBio Sequencing and Data Processing

The poly-A RNA from the root, rachis, and pinna of *A. spinulosa* was sequenced separately, yielding 20.83, 25.25, and 18.51 G subreads bases, respectively. Following self-correction, each organ generated 456,724, 457,745, and 431,767 CCS, of which 376,298, 397,732, and 357,877 corresponded to FLNC reads. The clustered FLNCs were refined with NFLNC reads to produce 41,012, 49,490, 45,454 HQ and 182,071, 173,240, 158,036 LQ polished consensus sequences for root, rachis, and pinna, respectively (Supplementary Table S1).

### Proofreading Full-Length Reads by Illumina Reads and *De Novo* Transcriptomes Assembly

For Illumina sequencing, the same poly-A RNA samples from root, rachis, and pinna were used, yielding raw reads of 9.18 G, 8.95 G, and 7.33 G, respectively (Supplementary Table S2). After quality control, the clean reads of 8.64 G, 8.67 G, and 6.72 G were applied to correct the polished consensus sequence. The corrected consensus sequences were represented by 223,083, 222,730, and 203,490 in the root, rachis, and pinna, respectively. Clean reads from three organs were independently assembled to 119,337, 59,933, and 62,387 unigenes with N50 of 1759 bp, 2027 bp, and 1893 bp, respectively (Table 1). A combination of clean reads from three organs was assembled to 168,042 unigenes with N50 of 2026 bp and 45.8% GC content (Table 1).

**TABLE 1:** Statistics for the assembled and the full-length transcriptomes

Sample	Unigenes type	Raw data (G)	<1 kb (%)	1–2 kb (%)	2–3 kb (%)	3–4 kb (%)	4–5 kb (%)	>5 kb (%)	Total number	N50 (bp)	GC content (%)
Root	Assembled	9.18	55.2	27.4	11.1	4.0	1.4	0.9	119,337	1759	46.0
	Full-length	20.83	8.0	16.0	22.0	17.0	19.7	17.4	125,758	4411	44.8
Rachis	Assembled	8.95	41.8	32.7	16.3	6.0	2.2	1.0	59,933	2027	46.0
	Full-length	25.25	10.1	17.9	20.0	21.8	21.2	8.9	89,107	3,944	46.3
Pinna	Assembled	7.33	45.0	32.7	15.4	4.7	1.6	0.6	62,387	1893	45.9
	Full-length	18.51	6.0	15.6	24.0	20.7	24.2	9.5	89,332	4005	45.6
Mixed *de novo* assembly		—	49.8	27.4	13.7	5.7	2.1	1.3	168,042	2026	45.8
Non-redundant reference transcriptome		—	8.0	15.7	21.7	19.5	22.1	13.1	278,357	4141	45.4

### Three-Organ Full-Length Transcriptomes and a Non-Redundant Reference Transcriptome

The corrected consensus sequences of root, rachis, and pinna were clustered into 125,758, 89,107, and 89,332 FL unigenes, respectively. The N50 of the FL unigenes of each organ was 4411 bp, 3,944 bp, and 4005 bp, with GC contents of 44.8, 46.2, and 45.6%, respectively (Table 1). FL unigenes from roots, rachis, and pinna were combined and clustered into a non-redundant FL reference transcriptome of *A. spinulosa.* There were 278,357 FL unigenes in the reference transcriptome, with N50 of 4141 bp (Table 1). The majority of assembled unigenes (41.8%–55.2%) were smaller than 1 kb in length, while the FL unigenes were enriched at two ranges: 2–3 kb (20.0%–24.0%) and 4–5 kb (19.7%–24.2%) (Figure 1B). The GC content of the FL transcriptome was 0.4% lower than the assembled transcriptome (Table 1).

### Alternative Splicing and Completeness Evaluation of Transcriptomes

Of the 1,614 benchmarking universal single-copy orthologs in embryophyte, 1,443 (89.4%) orthologs were completely detected in the mixed assembled transcriptome, while 1,490 (92.3%) orthologs were recognized in the FL reference transcriptome (Figure 1C). For the root, rachis, and pinna FL transcriptomes, 1,245 (77.1%), 1,275 (79.0%), and 1,286 (79.7%) of the 1,614 conserved orthologs were detected, respectively (Figure 1C). These results suggest that *A. spinulosa* FL transcriptomes are fairly intact and suitable for downstream analysis.

The 278,357 FL unigenes were reconstructed into 38,470 UniTransModel, which indicated that the multi-organ unigenes obtained by Iso-Seq pipeline and CD-HIT were still at a high level of redundancy. For root, rachis, and pinna, 58,707, 50,907, and 56,064 FL unigenes were aligned to 37,021, 28,784, and 30,317 UniTransModels, respectively (Supplementary Table S3). The UniTransModels with only one isoform accounted for 68.3%, 63.0%, and 62.6% of the root, rachis, and pinna. The number of isoforms in UniTransModels decreased gradually from 1 to 10 (Supplementary Table S3). In total, 6,841, 8,397, and 10,319 AS events were captured from root, rachis, and pinna (Figure 1D). The AS events were negatively correlated with the number of isoforms, where the highest proportion of single isoforms and the lowest number of AS events were found in root, while the lowest proportion of single isoforms and the highest number of AS events were found in pinna. RI was the most common AS event in three organs, followed by A3 and A5 events. The AL and MX events were the two least types. These results indicated AS events may be associated with genes carrying out different functions in three organs.

### Functional Annotation of Three-Organ Full-Length Transcriptomes

By annotating the FL transcriptome through six public databases, comprehensive functional information about the FL unigenes was obtained (Table 2, Supplementary Table S4). The annotation rates for the three organs were similar, with the NR database having the highest annotation rate (84.4%–90.1%) and the NT database having the lowest (31.4%–42.2%). There were 89.1%, 90.0%, and 92.8% of FL unigenes annotated in at least one database and 22.1%, 19.1%, and 21.4% of FL unigenes assigned in all databases for root, rachis, and pinna, respectively. In the non-redundant FL reference transcriptome, a total of 250,223 (89.9%) FL unigenes were annotated, with 57.159 (20.5%) FL genes captured in all databases.

**TABLE 2:** Statistics for the annotated results of FL transcriptomes in the public databases

Annotation	Root	Rachis	Pinna	Non-redundant reference
NT	53,119 (42.2%)	28,023 (31.4%)	31,899 (35.7%)	102,992 (37.0%)
NR	106,089 (84.4%)	76,105 (85.4%)	80,453 (90.1%)	238,100 (85.5%)
KOG	75,611 (60.1%)	54,938 (61.7%)	56,647 (63.4%)	170,030 (61.1%)
Swiss-Prot	94,324 (75.0%)	68,821 (77.2%)	72,171 (80.8%)	213,291 (76.6%)
Pfam & GO	70,177 (55.8%)	53,964 (60.6%)	55,294 (61.9%)	160,995 (57.8%)
KEGG	103,316 (82.2%)	74,517 (83.6%)	79,351 (88.8%)	232,864 (83.7%)
At least one database	112,058 (89.1%)	80,178 (90.0%)	82,877 (92.8%)	250,223 (89.9%)
All databases	27,752 (22.1%)	17,051 (19.1%)	19,134 (21.4%)	57,159 (20.5%)

**FIGURE 2:**
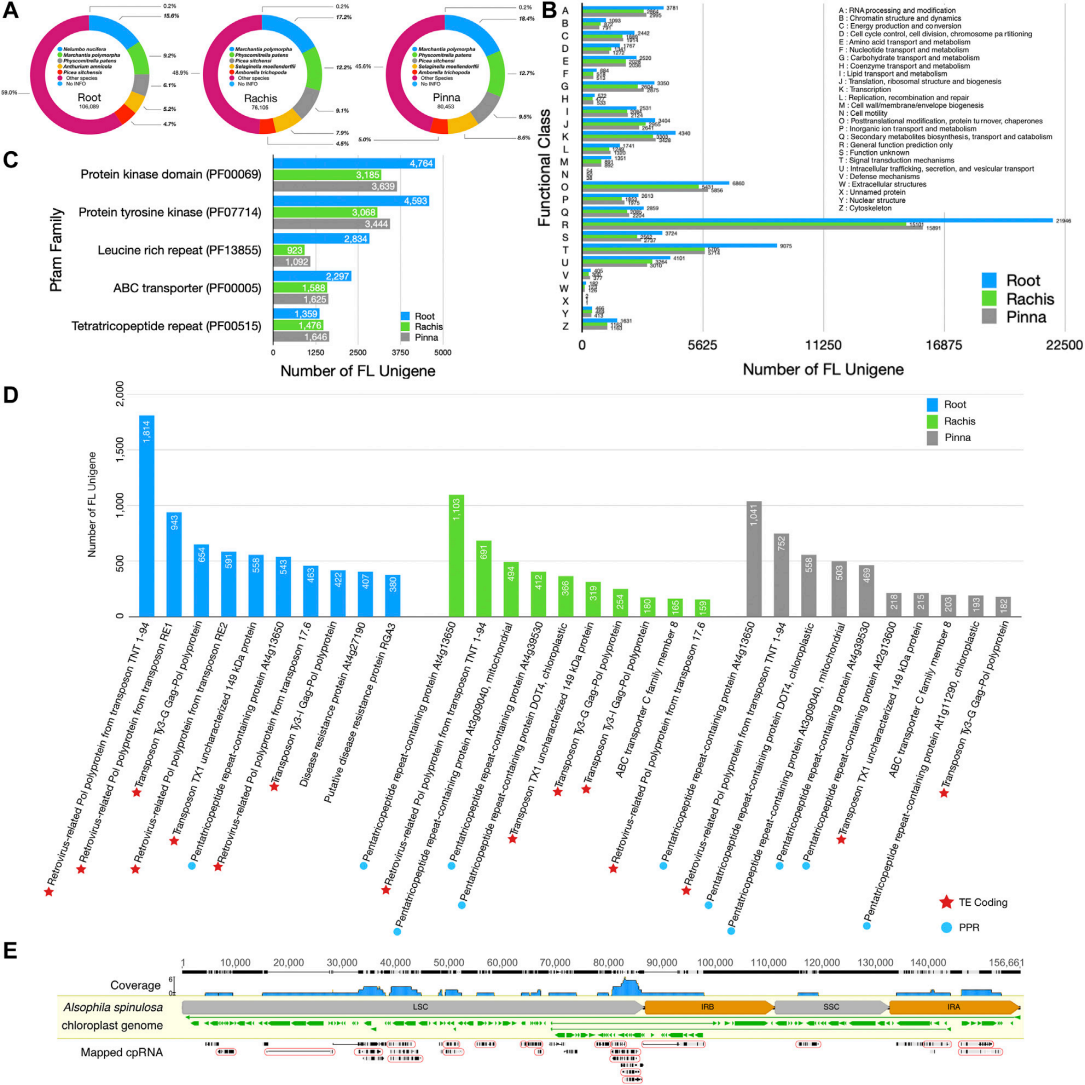
The NR, Pfam, KOG, and Swiss-Prot databases annotation statistics of three organ FL transcriptomes and cpRNA identification from NT database. **(A)** The top five species distribution of best BLASTX hits against the NR database. **(B)** KOG functional classification of FL unigenes. **(C)** The top five protein families annotated to each organ transcriptome in the Pfam database. **(D)** The top ten most annotated proteins per organ transcriptome in the Swiss-Prot database. **(E)** Results of mapping FL unigenes to the chloroplast genome. In the “mapped RNA” row, the grey and black bars indicate matching and mismatching with the chloroplast genome, respectively. The thin lines indicate the gap in the alignment. The mapped cpRNA in the circle are fusion transcripts.

Based on species information from the NR database, each of the three FL transcriptomes was substantially aligned to the sequences of *Marchantia polymorpha* (9.2% in root, 17.2% in rachis, 18.4% in pinna), *Physcomitrella patens* (6.1% in root, 12.2% in rachis, 12.7% in pinna), and *Picea sitchensis* (4.7% in root, 9.1% in rachis, 9.5% in pinna) (Figure 2A). Fern species were not included in the top five species aligned to the NR database, due to the lack of molecular resources of ferns. The KOG database classified the unigenes into 26 groups by function (Figure 2B), and the largest category assigned to each of the three organs FL unigenes was “general function prediction (R)” (21,946 in root, 15,101 in rachis, 15,891 in pinna). Next, “signal transduction mechanisms (T)” (9,075 in root, 5,705 in rachis, 5,714 in pinna) and “post-transcriptional modifications, protein turnover, chaperones (O)” (6,860 in root, 5,431 in rachis, 5,856 in pinna) were the second and third representative functional categories of FL unigenes, respectively. Among the FL unigenes annotated in the Pfam database, the two most abundant protein families were “protein kinase domain” and “protein tyrosine kinase” (Figure 2C). The “leucine rich repeat” (2,834 in root), “ABC transporter” (1,588 in rachis), and “tetratricopeptide repeat” (1,646 in pinna) protein families were the third-largest in the FL transcriptomes of root, rachis, and pinna, respectively.

Annotation statistics of FL unigenes in the Swiss-Prot database revealed that 1814 FL unigenes in the root transcriptome were mapped to “retrovirus-related Pol polyprotein from transposon TNT 1–94” (Figure 2D). The FL unigenes of root were also aligned to other transposon-encoded polyproteins, such as “retrovirus-related Pol polyprotein from transposon RE1” (943), “transposon Ty3-G Gag-Pol polyprotein” (654), and “retrovirus-related Pol polyprotein from transposon RE2” (591). In particular, the FL unigenes of root are rich in plant disease resistance proteins, such as “disease resistance protein At4g27190” (407) and “putative disease resistance protein RGA3” (380). The “pentatricopeptide repeat-containing protein At4g13650” was the most abundant unigenes in the rachis and pinna, with 1,103 and 1,041 hits, respectively. The FL unigenes of the rachis and pinna also hit additional types of pentatricopeptide repeat-containing proteins, such as “pentatricopeptide repeat-containing protein DOT4, chloroplastic” (366 in rachis, 558 in pinna), “pentatricopeptide repeat-containing protein At3g09040, mitochondrial” (494 in rachis, 503 in pinna), and “pentatricopeptide repeat-containing protein At4g39530” (412 in rachis, 469 in pinna).

A total of 54 HQ FL unigenes hit the *A. spinulosa* chloroplast genome of the NT database, of which 35 were strictly remapped back to the chloroplast genome, covering 90,147 bp of 156,661 bp (57.5% of genome) (Figure 2E). A total of 22 unigenes were regarded as the fusion transcripts that comprised two or more chloroplast genes, especially those with similar functions. Four photoreceptor II subunit genes (*psbJ, psbL, psbF, psbE*), for example, were co-transcribed as i1_HQ_c8140/f2p13/1,547; five ribosomal protein genes (*rpl14, rpl16, rps3, rpl22, rps19*) were co-transcribed as two distinct fusion transcripts, i4_HQ_c7330/f2p8/4815 and i4_HQ_c12475/f2p3/4386; and four rRNA and two tRNA genes (*rrn5, rrn4.5, rrn23, trnA-UGC, trnI-GAU, rrn16*) were co-transcribed as i7_HQ_c646/f46p0/7,458 (Supplementary Table S5).

### Structural Prediction of Three-Organ Full-Length Transcriptomes

In total, 126,959, 90,491, 91,258 coding sequences were predicted from 123,328 unigenes (98.1%), 87,682 unigenes (98.4%), 88,441 unigenes (99.0%) in root, rachis, and pinna, respectively. There were 8,097, 6,009, and 6,108 transcription factors predicted in the FL transcriptomes of root, rachis, and pinna, respectively (Supplementary Table S6). C3H was the largest family in both root and pinna transcriptomes, with 404 and 345 members, respectively, while C2H2 was the greatest family in the pinna transcriptome, with 503 members. In the transcriptomes of root, rachis, and pinna, 159,374, 89,212, and 104,485 SSR were identified, respectively, with mono-nucleotide repeat being the most common (Supplementary Table S7). A total of 5,745, 3,784, and 2,708 LncRNA were isolated from the root, rachis, and pinna FL unigenes, respectively, by combining four approaches (Supplementary Table S8). LncRNA obtained from FL unigenes of root, rachis, and pinna had average lengths of 1898 bp, 1,263 bp, and 1873 bp, respectively, which were considerably shorter than mRNA.

### Genes Expression Quantification and Differential Expression Genes

By mapping the clean short reads from each of the three organs to the non-redundant FL transcriptome, the FPKM values of 278,357 FL unigenes in three organs were obtained. There were 154,744, 118,707, and 108,836 FL unigenes mapped for the root, rachis, and pinna, respectively (Table 3). Among the mapped FL unigenes of three organs, 59,942 (38.7%), 46,021 (38.8%), and 34,344 (31.6%) were considered as non-expressed genes (FPKM ≤ 0.3), which were excluded in the subsequent differential expression analysis. Aside from that, the majority of FL unigenes have FPKM values ranging from 1 to 10, with 46,869 (root), 34,748 (rachis), and 35,949 (pinna), respectively.

**TABLE 3:** The expression characteristics of all 278,357 FL unigenes in three organs.

FPKM interval	Root	Rachis	Pinna
(0,0.3]	59,942 (38.7%)	46,021 (38.8%)	34,344 (31.6%)
(0.3–1]	34,821 (22.5%)	25,380 (21.4%)	25,129 (23.1%)
(1–10]	46,869 (30.3%)	34,748 (29.9%)	35,949 (33.0%)
(10–100]	11,836 (7.6%)	11,038 (9.3%)	11,807 (10.8%)
(100, +∞)	1,276 (0.8%)	1,520 (1.3%)	1,607 (1.5%)
Total mapped FL unigenes	154,744	118,707	108,836

**FIGURE 3:**
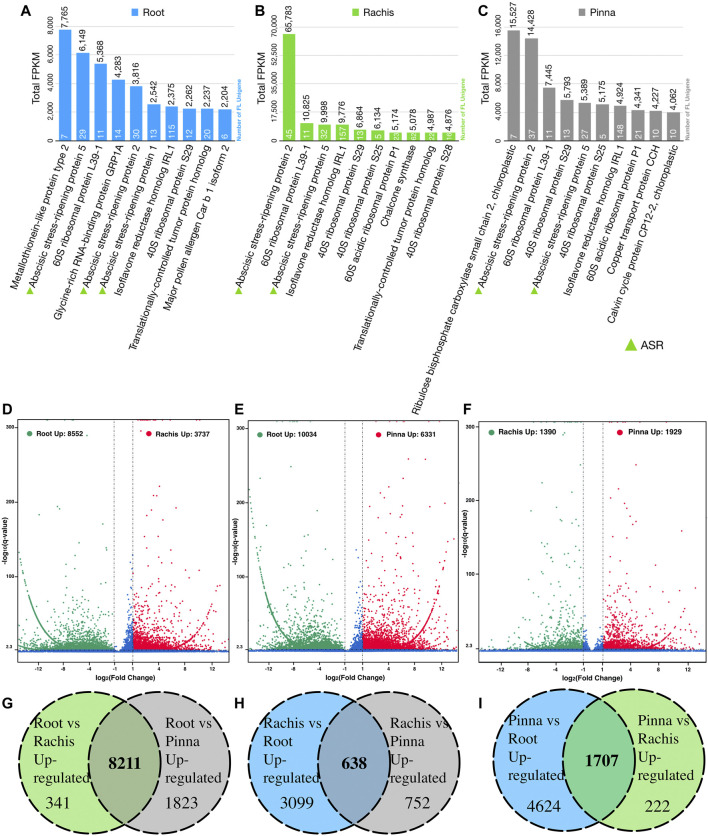
The top expressed genes in three organs based on Swiss-Prot database annotation and pairwise differential expression gene analysis. **(A)** The top ten expressed genes in root. **(B)** The top ten expressed genes in rachis. **(C)** The top ten expressed genes in pinna. **(D)** Rachis versus root differentially expressed genes. **(E)** Pinna versus root differentially expressed genes. **(F)** Pinna versus rachis differentially expressed genes. **(G)** Number of root upregulated genes versus rachis and pinna. **(H)** Number of rachis upregulated genes versus root and pinna. **(I)** Number of pinna upregulated genes versus root and rachis.

The top ten expressed genes in each of the three organs were determined by summing the FPKM values of FL unigenes that were assigned to the same protein in Swiss-Prot database (Figures 3A–C). The 60S and 40S ribosomal proteins were highly expressed across all three organs. More than 100 FL unigenes were annotated to “isoflavone reductase homolog IRL1” with remarkable FPKM values in all three organs. “Metallothionein-like protein type 2” was the highest expressed gene in the root transcriptome, although there was a very low expression in the rachis and pinna. “Ribulose bisphosphate carboxylase small chain 2, chloroplastic” was the most expressed gene in the pinna, which is barely expressed in the root. Surprisingly, three members of the abscisic stress-ripening protein family (*ASR1*, *ASR2*, and *ASR5*) were found in the highly expressed gene of root. Two members (*ASR2* and *ASR5*) were observed in rachis and pinna as well. The total FPKM value of *ASR2* gene in rachis reached 65,783.

Pairwise comparing the expressed FL unigenes (FPKM >0.3) between two organs, 8,552 and 10,034 unigenes were significantly upregulated in root versus rachis and pinna, respectively (Figure 3D, Supplementary Tables S9, S10); 3,737 and 1,390 unigenes were significantly upregulated in rachis versus root and pinna, respectively (Figure 3E, Supplementary Tables S9, S11); 6,331 and 1,929 unigenes were significantly upregulated in pinna versus root and rachis, respectively (Figure 3F, Supplementary Tables S10, S11). The FL transcriptome of the root, rachis, and pinna contained 8,211, 638, and 1707 unigenes that were upregulated against both other two organs, respectively (Figures 3G–I).

**FIGURE 4:**
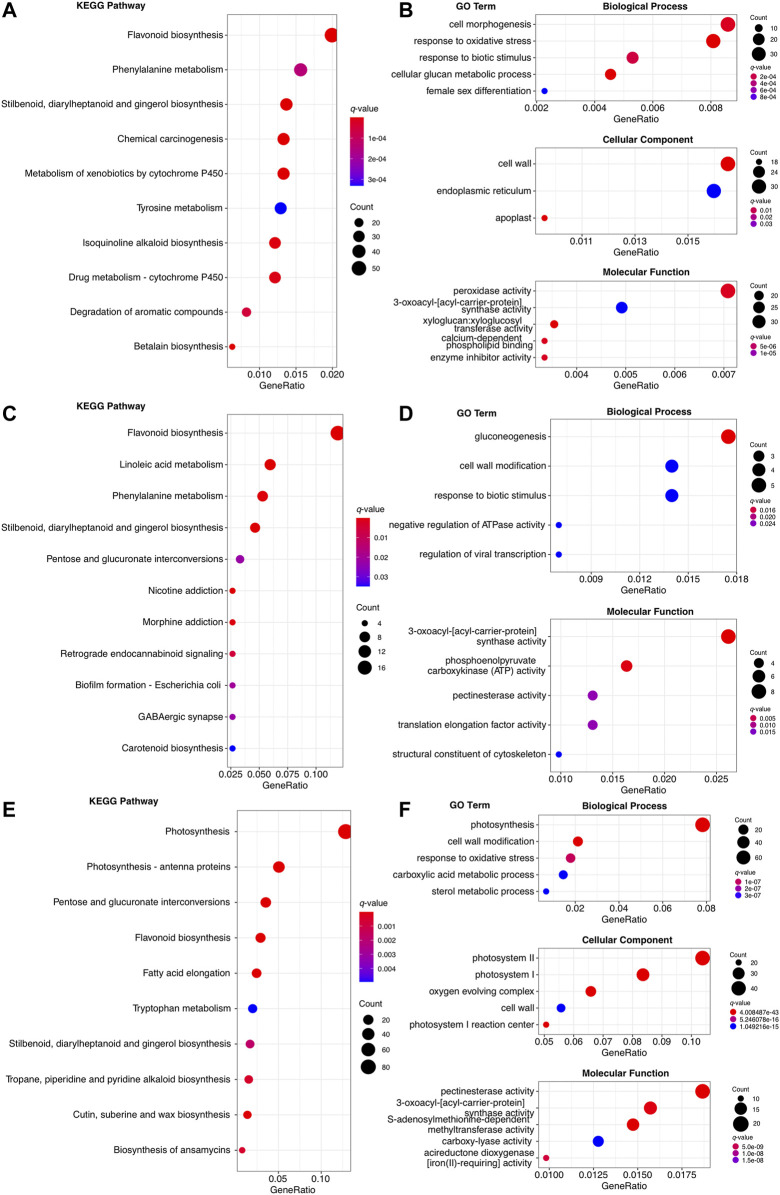
The KEGG and GO enrichment analysis of organ-specific upregulated genes. **(A)** The top ten enriched KEGG pathways in 8,211 upregulated genes of root. **(B)** The top ten enriched KEGG pathways in 638 upregulated genes of rachis. **(C)** The top ten enriched KEGG pathways in 1707 upregulated genes of pinna. **(D)** The top five enriched GO term of each level 1 category in 8,211 upregulated genes of root. **(E)** The top five enriched GO term of each level 1 category in 638 upregulated genes of rachis. None of the GO terms in the cellular component was enriched. **(F)** The top five enriched GO terms of each level 1 category in 1707 upregulated genes of pinna.

The 8,211, 638, and 1707 upregulated genes in root, rachis, and pinna were both enriched to the KEGG pathways related to secondary metabolism, such as “flavonoid biosynthesis” and “stilbenoid, diarylheptanoid, and gingerol biosynthesis,” as well as “response to biotic stimulus” GO term (Figures 4A–F). The 8,211 upregulated genes in root were specifically enriched for KEGG pathways associated with cytochromes P450 (Figure 4A), such as “metabolism of xenobiotics by cytochrome p450” and “drug metabolism - cytochrome P450”. The “phenylalanine metabolism,” “tyrosine metabolism,” and “betalain biosynthesis” KEGG pathways were also enriched in the upregulated genes of root, which were also associated with secondary metabolism. Root upregulated genes were enriched in the GO terms of “response to oxidative stress” and “peroxidase activity,” both of which were related to oxidative stress (Figure 4B). The 638 upregulated genes of rachis, besides being enriched to the common secondary metabolic KEGG pathways, were specifically enriched to the “linoleic acid metabolism” (Figure 4C). These upregulated genes in rachis were specifically enriched in the GO terms such as “gluconeogenesis” and “3-oxoacyl-acyl-carrier-protein synthase activity” (Figure 4D). KEGG and GO enrichment analysis revealed that 1707 upregulated genes in pinna were significantly involved in photosynthesis-related categories. These genes were enriched to the “photosynthesis” and “photosynthesis-antenna proteins” KEGG pathways and the “response to oxidative,” “photosystem” GO terms (Figures 4E,F). The upregulated genes in the pinna were specifically enriched to “fatty acid elongation” and “cutin, suberin, and wax biosynthesis” KEGG pathways. Since the root contained the highest number of upregulated genes, the results of its enrichment analysis were more significant. The results of the enrichment analysis in the rachis were less significant due to the lowest number of upregulated genes.

### Characterization of Photoreceptor Genes

**FIGURE 5:**
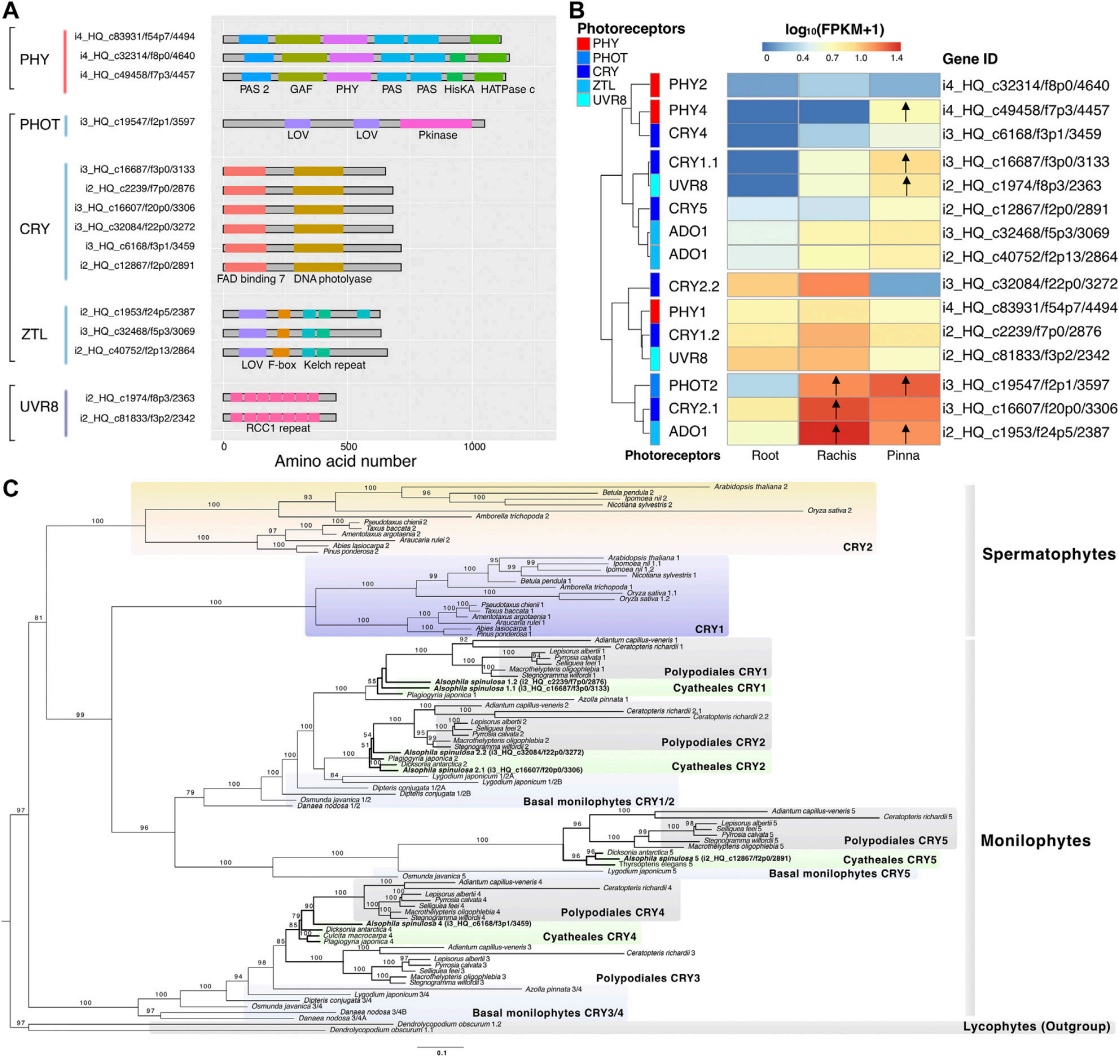
Characterization of photoreceptor genes and phylogenetic analysis of *CRY*. **(A)** The typical protein domain identified in five types of photoreceptors. **(B)** Heatmap represents the expression pattern of the photoreceptors in three organs. Arrows indicate the upregulated genes compared to their expression in the root. **(C)** The *CRY* phylogenetic tree of spermatophytes, monilophytes, and lycophytes constructed by maximum likelihood method. The number above the nodes indicates the bootstrap value, which was omitted if it was less than 50.

Based on the annotation of Swiss-Prot databases, 15 non-redundant high-quality photoreceptor genes were identified in the reference transcriptome of *A. spinulosa*, including 3 *PHY*, 1 *PHOT*, 6 *CRY*, 3 *ADO1* (ZTL family), and 2 *UVR8* (Supplementary Table S12). Neither was the *NEO* gene found in the best hits of all public databases, nor was the characteristic domain of *NEO* identified in Pfam database. The start and stop codons of each photoreceptor coding sequence were manually curated, after which these protein sequences were determined to contain the intact typical domains in the Pfam database (Figure 5A). *PHOT2* (i3_HQ_c19547/f2p1/3,597) and *ADO1* (i2_HQ_c1953/f24p5/2,387) were both upregulated in the rachis and pinna against its root expression (Figure 5B). *CRY2.1* (i3_HQ_c16607/f20p0/3,306) was upregulated in the rachis compared to its expression in root, while *PHY4* (i4_HQ_c49458/f7p3/4457), *CRY1.1* (i3_HQ_c16687/f3p0/3,133), and *UVR8* (i2_HQ_c1974/f8p3/2,363) were upregulated in the pinna versus its expression in root.

The phylogenetic tree of *CRY* revealed that *CRY* was divided into two branches (bootstrap value = 97), one containing the *CRY1/2* of spermatophytes and the *CRY1/2/5* of monilophytes, and the other containing the *CRY3/4* of monilophytes (Figure 5C). The species in Polypodiales, as the sister group of Cyatheales, had five members of *CRY* (1–5), each of which possessed one copy. The species of Cyatheales comprised a total of four members of *CRY* (1, 2, 4, 5), but different species covered distinct members. *A. spinulosa* covered four members of *CRY* (1, 2, 4, 5), while *Plagiogyria japonica* and *Dicksonia antarctica* covered three members of *CRY* (1, 2, 4) and *CRY* (2, 4, 5), respectively. Surprisingly, both *CRY* members 1 and 2 of *A. spinulosa* had two copies, whereas other Cyatheales species only had one.

### Identification and Validation of Abscisic Acid, Stress, and Ripening-Induced Gene Family

**FIGURE 6:**
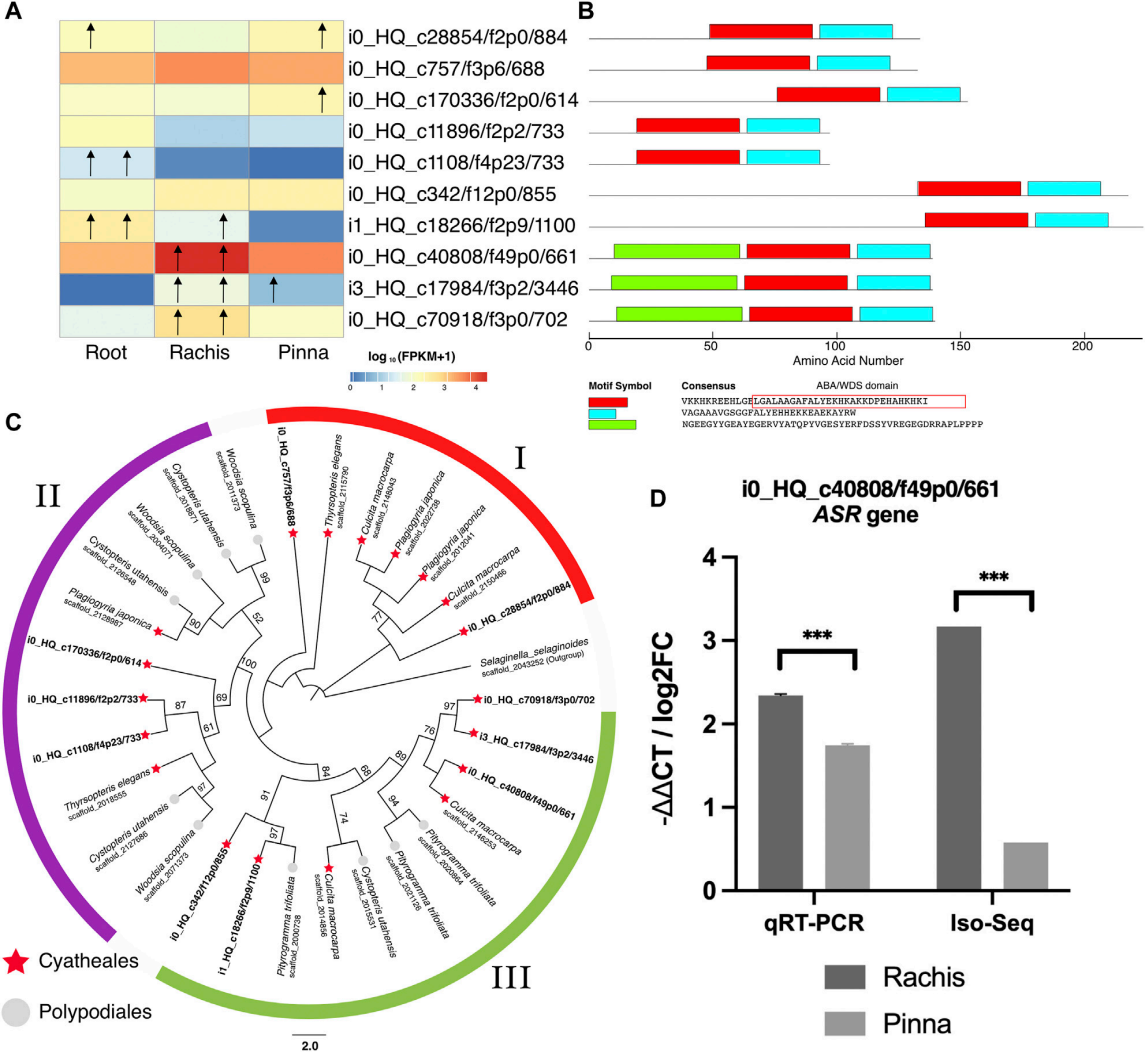
Identification and profiling of *ASR* gene family. **(A)** Expression profiling of the *ASR* genes family in three organs as a heatmap. Root column: left/right arrows indicate upregulated genes in root versus rachis/pinna. Rachis column: left/right arrows indicate upregulated genes in rachis versus root/pinna. Pinna column: left/right arrows indicate upregulated genes in pinna versus root/rachis. **(B)** The conserved motif identified in the *ASR* genes family. The amino acid residues in the rectangle indicated the ABA/WDS conserved domain. **(C)** Phylogenetic tree of Cyatheales and Polypodiales *ASR* genes family constructed by maximum likelihood. The number above the nodes indicates the bootstrap value, which was omitted if it was less than 50. **(D)** *ASR* FL unigene validation by RT-PCR.

Based on the annotation results of Swiss-Prot databases, 10 non-redundant *ASR* high-quality unigenes were identified in the reference transcriptome of *A. spinulosa*, with all members containing ABA-water deficit stress motifs (Figures 6A,B, Supplementary Table S13). By analyzing the expression profiles of the ten *ASR* members (Figure 6A), three members (i0_HQ_c40808/f49p0/661, i3_HQ_c17984/f3p2/3,446, i0_HQ_c70918/f3p0/702) were upregulated in the rachis versus root and pinna, all of which exclusively contained the yellow motif (Figure 6B). The phylogenetic tree of the *ASR* gene in Cyatheales and Polypodiales was loosely divided into three branches (Figure 6C, the bootstrap value of branch I < 50, the bootstrap value of branch II = 100, the bootstrap value of branch III = 84). Branch I only harbored the species in Cyatheales, but the internal branches had low bootstrap values (<50). Branch II encompasses three members of the *ASR* gene family in *A. spinulosa*. Branch III covers five members of the *ASR* family in *A. spinulosa*. Two *ASR* members of *A. spinulosa* (i0_HQ_c342/f12p0/855 and i1_HQ_c18266/f2p9/1,100) were clustered together, with the latter upregulated expression in roots versus two other organs. The other three *ASR* members mentioned above (i0_HQ_c40808/f49p0/661, i3_HQ_c17984/f3p2/3,446, and i0_HQ_c70918/f3p0/702), which were upregulated in the rachis, were also evolutionarily clustered together.

To validate the accuracy of the gene expression analysis, an *ASR* gene (i0_HQ_AS3Root_c40808/f49p0/661) was verified by qRT-PCR. Using the root as control sample, the expression pattern of the *ASR* gene in Iso-Seq analysis was correlated with the qRT-PCR assay (Figure 6D). Both were significantly expressed higher in the rachis than in the pinna. The results of qPR-PCR confirm the quantitative analysis of gene expression. The results of qPR-PCR experiment and the primer pairs are available in Supplementary Table S14.

### Stomatal Movement-Related Pathways Profiling

**FIGURE 7:**
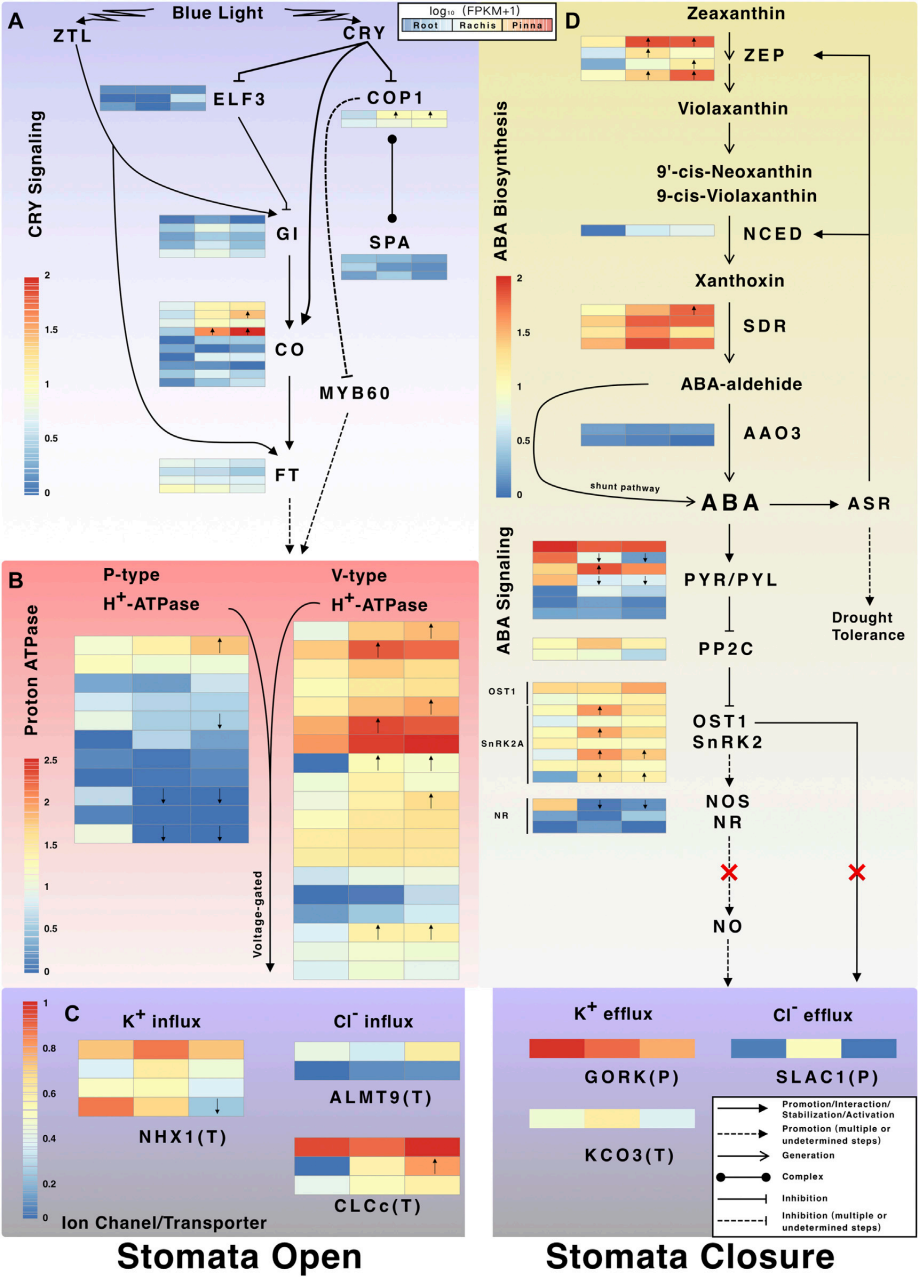
The CRY signaling pathway and ABA biosynthesis and signaling pathway. **(A)** Expression profiles of CRY-regulated stomatal opening-related genes. **(B)** Expression profiles of proton atpase genes; **(C)** Expression profiles of ion channel and transporter genes involved in stomatal movement. The P in parentheses indicates the ion channel/transporter located at the plasma membrane. The T in parentheses indicates the ion channel/transporter located at tonoplast. **(D)** Expression profiles of genes in ABA biosynthesis and signal transduction pathway. Arrows inside the heatmap indicate the up/downregulated genes in rachis/pinna versus root.

Three types of suppressor genes downstream of CRY were expressed at low levels, including E3 ubiquitin-protein ligase (*COP1*) that inhibits photomorphogenesis, suppressor of PHYA (*SPA*) that comprises the COP/SPA complex, and early flowering 3 (*ELF3*) that regulates the biological clock (Figure 7A). The reference transcriptome revealed three types of activator genes that stimulate stomatal opening downstream of CRY, including GIGANTEA (*GI*), CONSTANS (*CO*), and flowering locus T (*FT*). Two members of *CO* transcription factor (i1_HQ_c11655/f4p9/1,526 and i1_HQ_c2554/f7p0/1,441) were upregulated in pinna versus root (Figure 7A). The P-type and V-type proton atpase were identified, with the latter having more members in the pinna and rachis versus root (Figure 7B). The proton pump altered the electrical potential of plasma membrane/tonoplast and then activated the ion channels/transporters associated with stomatal opening, of which three types were discovered in the reference transcriptome (Figure 7C). Among the three types of ion channel/transporters (*NHX1*, sodium/hydrogen exchanger 1; *ALMT9*, aluminum-activated malate transporter 9; *CLCc*, chloride channel protein C), *CLCc* gene members exhibited high expression levels in the pinna (Supplementary Table S15).

The genes encoding the enzymes (*ZEP*, zeaxanthin epoxidase, chloroplastic; *NCED*, nine-*cis*-epoxycarotenoid dioxygenase; *SDR*, short-chain dehydrogenases/reductases) for the first three steps of ABA biosynthesis were expressed higher in pinna and rachis than root (Figure 7D). *NCED*, the rate-limiting enzyme for ABA synthesis, was expressed exclusively in pinna and rachis. The gene encoding abscisic-aldehyde oxidase 3 (*AAO3*), which catalyzes the final step of ABA synthesis, was expressed at very low levels across all organs. There is, however, a shunt pathway in plants that directly synthesizes ABA without enzyme catalysis. The genes encoding the pyrabactin resistance/pyr1-like/regulatory component of ABA receptor family (*PYR/PYL*) in the first step of ABA signal transduction pathway were expressed in all organs, but the members showed varied expression patterns (Figure 7D). The genes encoding *PP2C* (protein phosphatase 2C), a suppressor in the ABA signal transduction pathway, were expressed in trace levels in all organs. Two SnRK2-type genes were identified, including *OST1* (open stomata 1, also known as SnRK2.6) and *SnRK2A*. SnRK can promote NO (nitric oxide) synthesis, which requires a gene encoding nitric oxide synthase (*NOS*) which was not found in *A. spinulosa*, or another gene encoding nitrate reductase (*NR*) which was only weakly expressed in the rachis and pinna. Three types of genes encoding ion channels, guard cell outward rectifying K^+^ channel (*GORK*), Ca2^+^ activated outward-rectifying K^+^ channel 3 (*KCO3*), and *SLAC1* were discovered, which potentially induce stomatal closure (Figure 7C). The gene encoding *SLAC1*, a direct activation receptor for *OST1*, was almost not expressed in the pinna.

## Discussion

### Assessment of Full-Length Transcriptomes

Although there are two assembled transcriptomes of *A. spinulosa* (Dong et al., 2019; One Thousand Plant Transcriptomes Initiative et al., 2019), high-quality transcriptomes of ferns, especially for tree fern, are extremely scarce. In this study, 125,758, 89,107, and 89,332 FL unigenes were generated from the roots, rachis, and pinna of *A. spinulosa*, respectively. The unigenes from three organs were combined to the non-redundant reference transcriptome with 278,357 unigenes and N50 of 4,141 bp. The number of unigenes in the reference transcriptome was 4.5-fold and 4.0-fold greater and the N50 was 2.3-fold and 3.6-fold higher than that of the single-organ transcriptome reported from Dong et al. (2019) and OneKP database (2019), respectively. In this study, a total of 1,697,651 reads from three organs of *A. spinulosa* were reconstructed into 38,470 UniTransModels. For comparison, around 850,000 sequencing reads of *C. richardii* with a genome size of 11.25 Gb were reconstructed into 18,179 UniCFernModels by the same protocol (Marchant et al., 2019). These results indicated that genome size did not correlate with transcriptome size and more genes were covered by multi-organ sequencing than by single organ sequencing. Similarly, the novel strategy of multi-organ FL transcriptome for gene analysis has been used for several non-model plants, including *Asarum sieboldii* (Chen et al., 2020), *Pseudotaxus chienii* (Liu et al., 2020), and *Cephalotaxus oliveri* (He et al., 2021).

### Large Gene Families and Highly Expressed Genes

To gain insight into *A. spinulosa*, the FL transcriptome was decoded by functional annotation and structural prediction. In each of the three organs transcriptomes, approximately 90% unigenes were annotated to functional genes, as well as 3.0%–4.6% unigenes were predicted as lncRNA, indicating the FL transcriptomes were informative. Leucine-rich repeats (LLRs) are one of the largest gene families in the root transcriptome. Correspondingly, the root transcriptome is particularly abundant in disease resistance proteins, which typically contain the LLR domains for protein-protein interactions (Martin et al., 2003). Under biotic stress, plants deploy effectors triggered immunity (ETI) and induce disease resistance proteins for recognition of pathogen effectors. It implies that, as an underground organ, the root is more likely to be attacked by pathogens than rachis and pinna, leading to a higher abundance of disease-resistant protein. Transposon-encoded polyproteins, which were also related to biotic and abiotic stress resistance, were abundant in each of the organ transcriptomes, especially in root. A total of 10,142 FL unigenes were annotated as transposon-encoding protein genes in the Swiss-Prot database, accounting for 3.64% of the reference transcriptome, of which 8,977 were assigned to polyprotein. LTR transposon transposition is mediated by polyproteins (Grandbastien et al., 1989), implying that LTR transposons are potentially active in *A. spinulosa*. The transpositional activation of LTR transposon seems to increase plant resistance to stress (Grandbastien, 1998) and genome size. Coincidentally, 24.56% of the genome of *C. richardii*, a polypod fern, consists of LTR transposons (Marchant et al., 2019). This study found numerous polyprotein transcripts in *A. spinulosa*; however, the mobility of the LTR transposons at the genome level has yet to be demonstrated.

Based on Swiss-Prot database annotation, the largest gene family in the reference transcriptome is *PPR*, with 12,002 members and accounting for 4.3% of the total. The *PPR* gene family is responsible for precise post-transcriptional processing and expression regulation of chloroplast genes (Manna, 2015), which play an important role in plant response to abiotic stressors (Dinh et al., 2016). The transcriptome of pinna was the most abundant in *PPR* genes, reflecting varied post-transcriptional processing in the photosynthetic organs. Similarly, the largest gene family in the heterosporous fern genome is the *PPR* family, which is involved in the extensive organelle RNA processing of fern (Li et al., 2018). The fern chloroplast genome contains various post-transcriptional modifications (Wolf et al., 2004), among which RNA editing has been reported in *A. spinulosa* (Gao et al., 2009). In this study, the actual transcription of the chloroplast genome of *A. spinulosa* was revealed based on the reference transcriptome. Mapping the HQ FL unigenes back to the chloroplast genome of *A. spinulosa* revealed 22 polycistronic transcripts. Function-related gene clusters on the chloroplast genome, such as rRNA (*rrn5, rrn4.5, rrn23, trnA-UGC, trnI-GAU, rrn16*), ribosome protein (*rpl14, rpl16, rps3, rpl22, rps19*), photosystem II (*psbJ, psbL, psbF, psbE*), and NADH-plastoquinone oxidoreductase (*ndhJ, ndhK, ndhC*), were co-transcribed. The polycistronic transcript containing four photosystem II genes was also found in the chloroplast genome of *Adiantum capillus-veneris* (Wolf et al., 2004)*.* Short-read sequencing revealed that *rpl36* and *infA* were co-transcribed in *Adiantum capillus-veneris* (Wolf et al., 2004), while long-read sequencing unveiled that *rpoA*, *rps11*, *rpl36*, and *infA* were co-transcribed in *A. spinulosa*. Compared to short-read sequencing, long-read sequencing may offer additional information about the fusion cpRNA.

The highly expressed genes were obtained based on the annotation of Swiss-Prot database and the FPKM value of each organ transcriptome. Strikingly, the *ASR* genes induced by water deficiency, salt, cold, osmotic pressure, and ABA stress were substantially expressed across three organs (Maskin et al., 2001). Overexpression of the *ASR* gene in tomato boosted its drought tolerance (Golan et al., 2014). *Alsophila spinulosa* inhabits moist surroundings (Zhiying et al., 1990), and the high expression of *ASR* genes may improve its water retention ability. The expression of *ASR* genes was the highest in rachis among all three organs studied; however, the potential functions of *ASR* genes in *A. spinulosa* must be confirmed by further molecular and physiological research. A total of 10 non-redundant HQ FL unigenes annotated as *ASR* in Swiss-Prot database were identified in *A. spinulosa*. The *ASR* genes of *A. spinulosa* contain the characteristic ABA/WDS domain, as those of angiosperms (Henry et al., 2011). The *ASR* genes of ferns are roughly divided into three branches, according to phylogenetic analysis. The number of *ASR* genes varies between fern species, and the phylogenetic tree of *ASR* genes does not coincide with the phylogenetic tree of species (PPG I, 2016), which is similar to the large-scale phylogenetic analysis of *ASR* genes in angiosperms (Li et al., 2020). Notably, the reference transcriptome across all three organs contained a large number of genes attributed to “Isoflavone reductase homolog IRL1” with high expression in total. The IRL protein is most likely implicated in secondary metabolism in response to biotic and abiotic stressors (Shoji et al., 2002). In addition, the IRL protein regulates the lignin content, which provides mechanical support for higher plants (Hua et al., 2013). However, further investigation into the role of *IRL* genes in tree fern species is required. Overall, the large gene families and the highly expressed genes in the FL transcriptome of *A. spinulosa* are primarily related to stress response.

### Organ-Specific Up-Regulated Genes

When compared to the other two organs, the root transcriptome had the most upregulated genes, followed by pinna, and rachis had the least. The upregulated genes in each organ are enriched to the secondary metabolism pathways in KEGG database. The “flavonoid biosynthesis” pathway is enriched in three organs of upregulated genes together. Correspondingly, a variety of flavonoids have been isolated from *A. spinulosa* (Chiang et al., 1994). Flavonoids are associated with biotic stress responses in plants (Padmavati et al., 1997). Besides, the upregulated genes are enriched to other secondary metabolite pathways, such as “stilbenoid, diarylheptanoid and gingerol biosynthesis” (in root, rachis, and pinna), “phenylpropanoid biosynthesis” (in root and rachis), “betalain biosynthesis” (in root), and “tropane, piperidine and pyridine alkaloid biosynthesis” (in pinna). It shows that secondary metabolites play a crucial role across three organs, but their functions need to be studied further.

The upregulated genes of root, in particular, are enriched to the cytochrome P450-related KEGG pathway. The cytochrome P450 of plants is responsible for xenobiotic detoxification (Robineau et al., 1998). In agreement with the finding above, the roots are subjected to more exogenous stress, leading to an upregulation of the resistance response pathway as compared to the other two organs. The upregulated genes of pinna are enriched to photosynthesis-related categories both in KEGG pathway and GO terms, as expected.

### Characterization of Photoreceptor Genes and CRY Signaling Pathway

The evolution of light adaptation is a popular research field in ferns, especially the photoreceptor genes (Li et al., 2015a; Cai et al., 2020). In this study, the transcripts of photoreceptor genes of a tree fern species are completely characterized for the first time. In the reference transcriptome of *A. spinulosa*, 15 non-redundant photoreceptor genes were identified. *Alsophila spinulosa* contains three *PHY* genes, *PHY1*, *PHY2*, and *PHY4*, which are identical to the report in *Adiantum capillus-veneris* (Li et al., 2015a). The *NEO* gene, previously named *PHY3*, was found in *Adiantum capillus-veneris* (Kanegae et al., 2006) and *Plagiogyria distinctissima* (Yang et al., 2010), a non-tree fern species in Cyatheales. However, the *NEO* gene is neither reported for tree fern species nor is it found in the reference transcriptome of *A. spinulosa*. The *PHY* genes perhaps act redundantly in *C. richardii* in red light perception (Bissoondial, 2005), whereas the three *PHY* genes of *A. spinulosa* are expressed differentially throughout three organs. *PHY4* is specifically upregulated in the pinna, which may be the primary red-light receptor for *A. spinulosa* in its native habitat. Only the *PHOT2* gene is found in *A. spinulosa*, while *PHOT1* and *PHOT2* were detected in the species of heterosporous fern and polypod fern (Li et al., 2015b). Among the five types of photoreceptors in *A. spinulosa*, *CRY* genes are the most abundant type with six members. Following the phylogenetic tree of *CRY* genes in ferns inferred by Cai et al. (2020), the six members of *CRY* are classified as two *CRY1*, two *CRY2*, one *CRY4*, and one *CRY5*. As reported by Cai et al. (2020), the basal monilophytes contain *CRY1/2*, *CRY3/4*, and *CRY5*, and the most evolved taxon, polypodiales, contains *CRY1*, *CRY2*, *CRY3*, *CRY4*, and *CRY5*. Combining the reference transcriptome of *A. spinulosa* and the OneKP database, only *CRY1*, *CRY2*, *CRY4*, and *CRY5* are found in Cyatheales. The *CRY4* gene of *C. richardii* was upregulated under blue light treatment (Cai et al., 2020) and the CRY4 photoreceptor of *Adiantum capillus-veneris* was localized in the nucleus (Imaizumi et al., 2000), suggesting that CRY4 may be a photoreceptor for expression modulation in blue light. In this study, *CRY4* gene of *A. spinulosa* is found to be restricted to the rachis and pinna under natural conditions. Among the *CRY* members of *A. spinulosa*, only *CRY1.1* is upregulated in pinna versus root. Further physiological studies are needed to determine whether the numerous *CRY* genes in *A. spinulosa* are related to light adaptation, as in polypod ferns (Cai et al., 2020).

Cai et al. (2020) proposed that the rapid opening of stomata under blue light is tightly related to the light adaptation of understory ferns. In this study, three activator genes, *GI*, *GO*, and *FT*, of the CRY signaling pathway that indirectly induced stomatal opening were identified in *A. spinulosa*. Notably, the *FT* gene is not present in *A. spinulosa*, but its homolog gene, protein mother of FT and TFL1 (*MFT*), is found. Likewise, in *Pteridium aquilinum*, a polypod fern, only the *MFT* genes are identified (Karlgren et al., 2011). FT activates the P-type and V-type proton pump on guard cells through a series of downstream regulations, which are key components in the regulation of stomatal opening (Kinoshita and Hayashi, 2011). The proton pump further alters the membrane potential and activates a variety of ion channels/transporters in the tonoplast that promote stomatal opening. Among them, *NHX1*, *ALMT9*, and *CLCc* are detected in the reference transcriptome of *A. spinulosa*. *CLCc* genes, which encode a voltage-gated chloride channel, had relatively high expression in the pinna. CLCc is responsible for the accumulation of Cl^−^ in vacuole, which involves osmoregulation and stomatal opening (Jossier et al., 2010). This is the first work to describe the transcripts in CRY signaling pathway in *A. spinulosa* under natural conditions, which provides a valuable resource for future research on light adaptation of tree ferns (Large and Braggins, 2004).

### Characterization of Abscisic Acid Biosynthesis and Signaling Pathway

The active stomatal control to improve water-use efficiency is regarded as a crucial step in the evolution of angiosperms (Brodribb and McAdam, 2011). Therefore, the differences in stomatal closure mechanism between angiosperms and ferns, as the early-diverging and second-largest vascular plant lineage, are a hot research topic. Most ferns, including *Pteridium esculentum* (Brodribb and McAdam, 2011) and *Dicksonia antarctica* (McAdam and Brodribb, 2012), use the passive (hydraulic-mediated) mechanism to close their stomata. In contrast, the active (ABA-mediated) mechanism is documented in two fern species, *Polystichum proliferum* and *N. exaltata* (Cai et al., 2017). To date, whether the presence of ABA-mediated stomatal closure mechanism in ferns remains controversial. Based on transcriptomic data, the present study characterizes the transcripts in ABA biosynthesis and signal transduction pathway of the tree fern species for the first time. The reference transcriptome of *A. spinulosa* covers the entire biosynthesis pathway from zeaxanthin to abscisic (ABA)-aldehyde; especially, several *ZEP* and *SDR* members are upregulated in rachis and pinna versus root. The gene encoding the rate-limiting enzyme *NCED* was only expressed in rachis and pinna, indicating that ABA synthesis occurs exclusively in these two organs. ABA-aldehyde, the ABA precursor, is catalyzed by AAO3 to form ABA; however, its expression was very low across all organs. It has been discovered that in plants, ABA can be produced from ABA-aldehyde via a shunt pathway without enzyme (Rock et al., 1991), which may be the case for the final step of ABA biosynthesis in *A. spinulosa*. The highly expressed *ASR* genes are induced by ABA stress and positive feedback to promote ABA biosynthesis (Li et al., 2017).

The ABA signaling pathway consisting of PYR/PYL, PP2Cs, and SnRK2 is evolutionarily conserved in terrestrial plants (Hauser et al., 2011), which are also completely identified in the reference transcriptome of *A. spinulosa*. In seed plants, OST1, also known as SnRK2.6, directly activates SLAC1 anion efflux channels in guard cells to actively control stomatal closure (Geiger et al., 2009). Two genes encoding *OST1* are detected in three organs of *A. spinulosa*; however, the gene encoding *SLAC1* was almost not expressed in the pinna where the guard cell is located. The OST1-SLAC1 pathway associated with active stomatal closure may not be present in *A. spinulosa,* in agreement with the previous finding that this pathway might not be universal in all land plants (Cai et al., 2017). Alternatively, in angiosperms, the pathway downstream of SnRK2 activates the ion channels involved in stomatal closure by producing NO, a signal factor (Gong et al., 2021). However, the *NOS* (Neill et al., 2002) and *NR* (Bright et al., 2006) genes for NO production are not identified and lowly expressed across three organs, respectively. Genetic characterization of *A. spinulosa* indicates that SnRK2 is likely unable to induce the NO signaling molecules due to the lack of transcripts encoding NO synthases. This finding is consistent with physiological studies in a batch of ferns, in which the SnRK2 pathway is incapable to synthesize NO and thus cannot activate the ion channel (Gong et al., 2021). Hence there is no ABA-induced active stomatal closure in ferns. Overall, the reference transcriptome supports the hypothesis that the transcripts associated with stomatal closure downstream of the SnRK2 pathway are deficient in *A. spinulosa*. The absence of ABA-induced mechanism for active stomatal closure in ferns might maximize the photosynthetic capacity under sunfleck conditions (Deans et al., 2019).

## Conclusion

In this study, the first FL transcriptome of the tree fern species, *A. spinulosa*, was reported by combining PacBio and Illumina sequencing. The FL transcriptomes of pinna, rachis, and pinna contain 125,758, 89,107, and 89,332 unigenes, respectively. Combining FL unigenes from the three organ transcriptomes yielded a non-redundant transcriptome with 278,357 unigenes, and N50 of 4141 bp was obtained. The FL non-redundant transcriptome was further reconstructed into 38,470 UniTransModels, based on which 6,841, 8,397, and 10,319 AS events were detected in root, rachis, and pinna, respectively. Functional annotation reveals that retrotransposon-encoded polyprotein genes are the most abundant unigenes in root, while *PPR* genes are the most abundant unigenes in pinna. A total of 22 unigenes are regarded as the fusion transcripts of the chloroplast genome. These two types of genes, which are extensive in *A. spinulosa*, are both associated with resistance to biotic and abiotic stress. Quantification of FL unigene expression revealed that *ASR* genes, involved in stress responses such as drought and ABA, are remarkably expressed in *A. spinulosa*, which are validated by qRT-PCR. The secondary metabolite pathways are enriched for three organ-specific upregulated genes. The genes of five photoreceptors, CRY signaling pathway, ABA biosynthesis and transduction pathway, and stomatal movement-related ion channel/transporter are characterized from the HQ FL unigenes. The characteristics of these genes are consistent with the previous physiological studies on ferns. The detailed analysis of *A. spinulosa* transcriptome yields valuable adaptation-related genetic resources for the relict tree fern. The comprehensive FL transcriptome data of *A. spinulosa* will facilitate molecular biology, adaptive evolution, and phylogenetic research of tree fern species.

## Data Availability

The raw reads of RNA-Seq were deposited in the SRA database as follows: SRR14672414 (root); SRR14672413 (rachis); SRR14672412 (pinna). The subreads BAM file of Iso-Seq can retrieve from the SRA database: SRR14674205 (root); SRR14674204 (rachis); SRR14674203 (pinna).

## Supplementary Material

The Supplementary Material for this article can be found online at: https://www.frontiersin.org/articles/10.3389/fgene.2021.784546/full#supplementary-material. The transcriptome data, UniTransModels, CDS sequences, function annotation, expression qualification, and the sequences used in phylogenetic analysis can be download from https://figshare.com/projects/Multi-organ_Full-length_Transcriptome_of_Tree_Fern_Alsophila_spinulosa/116511.
